# Comprehensive analysis of mRNA–microRNA–lncRNA expression profiles in post-traumatic elbow heterotopic ossification using RNA sequencing and experimental validation

**DOI:** 10.1080/07853890.2025.2611612

**Published:** 2026-01-13

**Authors:** Limin Wang, Fanxiao Liu, Lianxin Li, Nan Liu, Jinlei Dong

**Affiliations:** Department of Orthopaedics, Shandong Provincial Hospital affiliated to Shandong First Medical University, Jinan, Shandong, China

**Keywords:** Elbow trauma, expression profile, heterotopic ossification, high-throughput RNA sequencing

## Abstract

**Background:**

This study aimed to profile the molecular signatures of post-traumatic elbow heterotopic ossification (HO) to identify key regulators and potential therapeutic targets.

**Methods:**

Total RNA from post-traumatic elbow HO tissues (n=4) and normal bone tissues (n=6) was subjected to high-throughput sequencing to identify differentially expressed mRNAs (DEGs), microRNAs (DEMs), and lncRNAs (DELs). Bioinformatics analyses included Gene Ontology (GO), Kyoto Encyclopedia of Genes and Genomes (KEGG) pathway enrichment, protein-protein interaction network construction, and transcription factor (TF)-microRNA-mRNA network analysis. The expression trends of four most upregulated and four most downregulated DEGs were validated by real-time quantitative reverse transcription polymerase chain reaction (qRT-PCR).

**Results:**

We identified 2,138 DEGs, 40 DEMs, and 905 DELs. DEGs were significantly enriched in biological process “bone mineralization,” cellular component “plasma membrane,” molecular function “integrin binding,” and pathways including PI3K–Akt, NF-κB, JAK–STAT, and TNF signaling pathways. Hub genes with high connectivity included MMP9, IL6, MMP3, CTSK, and BGLAP. Integrated network analysis highlighted the transcription factor JUN and key microRNAs (hsa-miR-124-3p, hsa-miR-548c-3p, and hsa-miR-135b). The qRT-PCR results confirmed the expression trends of selected DEGs.

**Conclusions:**

This study, for the first time, profiled the differentially expressed mRNAs, microRNAs, and lncRNAs in post-traumatic elbow HO using high-throughput RNA sequencing. These findings provide valuable insights into the molecular mechanisms of HO following elbow trauma. The identified hub genes (MMP9, IL6, MMP3, CTSK, and BGLAP), key TF (JUN), and key microRNAs (hsa-miR-124-3p, hsa-miR-548c-3p, and hsa-miR-135b) may serve as potential therapeutic targets for preventing and treating post-traumatic elbow HO.

## Key Messages

1. This study, for the first time, profiled the differentially expressed mRNAs, microRNAs, and lncRNAs in post-traumatic elbow HO using high-throughput RNA sequencing.
2. These findings provide valuable insights into the molecular mechanisms of HO following elbow trauma.
3. The identified hub genes (MMP9, IL6, MMP3, CTSK, and BGLAP), key microRNAs (hsa-miR-124-3p, hsa-miR-548c-3p, and hsa-miR-135b), and key transcription factor (JUN) may serve as potential therapeutic targets for the prevention and treatment of post-traumatic elbow HO.

## Introduction

Heterotopic ossification (HO) is a pathological condition characterized by the formation of mature lamellar bone in non-skeletal tissues such as muscles, ligaments, or tendons. HO can be either congenital or acquired, with the elbow joint being one of the most frequently affected sites in acquired cases [1]. Previous studies have reported that, the incidence of post-traumatic elbow HO can reach up to 40% [1–4]. Following elbow trauma, injury to local bone and soft tissues, along with hematoma formation, may lead to the aggregation of multipotent mesenchymal stem cells capable of proliferating and differentiating into osteogenic, chondrogenic, myogenic, or fibrotic lineages. However, the specific regulatory mechanisms that drive mesenchymal stem cells to differentiate into cartilage and bone within non-skeletal tissues remain poorly understood [5,6].

In the early stage of elbow HO, patients may present with local fever, redness, swelling, and tenderness—symptoms that are often difficult to distinguish from post-traumatic inflammation. As the disease progresses, these symptoms may subside or resolve; however, the range of motion in the elbow joint typically decreases over time, potentially resulting in bony ankylosis. In addition, HO often coexists with joint capsule contracture, which further exacerbates the restriction in elbow mobility. In some cases, the ectopic bone may compress surrounding neurovascular structures, such as the ulnar, median, or radial nerves, leading to additional clinical manifestations [7]. Consequently, post-traumatic elbow HO severely impairs patients’ daily functioning and increases healthcare costs [8].

Currently, the molecular mechanisms underlying post-traumatic elbow HO remain poorly understood, limiting the development of effective preventive and therapeutic strategies. Nonsteroidal anti-inflammatory drugs (NSAIDs) have been shown to inhibit the osteogenic differentiation of progenitor cells and thereby prevent HO [9,10]. Although several studies have demonstrated that NSAIDs effectively reduce the incidence and severity of hip HO, their efficacy in post-traumatic elbow HO has not been well established. Furthermore, NSAIDs may impair bone formation and fracture healing, increasing the risk of nonunion and thus restricting their clinical application [11]. Low-dose radiation therapy has proven effective in preventing HO after total hip arthroplasty [12–14]; however, its role in post-traumatic elbow HO remains uncertain. Additionally, potential complications—including carcinogenic risks, progressive soft tissue contracture, delayed wound healing, and impaired fracture union—must be carefully considered. Surgical resection is currently regarded as an effective treatment for post-traumatic elbow HO; however, this approach carries a high recurrence rate (radiological: 82–100%; clinical: 17–58%) [15–17] and is associated with complications such as nerve damage, infection, and delayed wound healing.

The integration of RNA sequencing (RNA-seq) technology with bioinformatics analysis has been widely applied for large-scale identification of differentially expressed mRNAs, microRNAs, and long noncoding RNAs (lncRNAs) across various physiological and pathological conditions [18]. High-throughput transcriptomic analysis of post-traumatic elbow HO may help elucidate key regulatory molecules and mechanisms, providing critical insights for future clinical research. However, to date, no high-throughput sequencing studies have been conducted on post-traumatic elbow HO.

After elbow joint trauma, HO and joint capsule contracture frequently occur concurrently, together leading to post-traumatic elbow stiffness. Our research team has long been committed to investigating the molecular mechanisms as well as the clinical diagnosis and management of elbow stiffness [19,20]. Using global transcriptome sequencing technology, we were the first to explore the molecular mechanisms underlying joint capsule contracture associated with post-traumatic elbow stiffness in humans [21]. Therefore, in this study, we comprehensively analyzed the expression profiles of mRNAs, microRNAs, and lncRNAs in post-traumatic elbow HO and normal bone tissues using high-throughput RNA-seq. Functional enrichment analyses, including Gene Ontology (GO) and Kyoto Encyclopedia of Genes and Genomes (KEGG) pathway analyses, were conducted to annotate the biological functions of the key differentially expressed genes (DEGs) and to identify associated signaling pathways. Additionally, a protein–protein interaction (PPI) network was constructed to identify hub genes, while key transcription factors (TFs) and microRNAs were predicted through the construction of a TF–microRNA–mRNA regulatory network. This study aims to elucidate the molecular mechanisms and biological pathways involved in post-traumatic elbow HO, deepen our understanding of its onset and progression, and provide novel molecular targets for therapeutic intervention.

## Materials and methods

### Research cohort

The Ethics Committee of Shandong Provincial Hospital Affiliated to Shandong First Medical University gave its approval for this study. Written informed permission was acquired by each subject. Ten individuals who underwent elbow surgery were included. Patients undergoing elbow stiffness release surgery for radiographically verified, severe HO resulting from previous elbow trauma (fracture/dislocation) made up the HO group. Patients without a history of HO who were having surgical procedures for acute elbow fractures made up the Normal Control (NC) group. Age under 18, pregnancy, a diagnosis of autoimmune arthritis or cancer, and refusal to provide permission were the exclusion criteria. We collected standard demographic (sex, age, BMI) and clinical (affected side, HO grade per Hastings and Graham [22]) data for all subjects.

### RNA extraction from bone and HO tissue

Control bone fragments and surgically extracted post-traumatic HO tissues were snap-frozen in liquid nitrogen. A mechanical homogenizer was used to homogenize around 30 mg of tissue in 350 µL of TRIzol Reagent (Invitrogen) in order to extract RNA. The aqueous layer was mixed with an equivalent amount of 70% ethanol after phase separation with chloroform. This mixture was then subjected to purification through an RNeasy Mini spin column (Qiagen), following the kit’s instructions precisely. The purified RNA was finally eluted in 12 µL of RNase-free water. We assessed the integrity of all RNA samples on an Agilent 2100 Bioanalyzer, proceeding to sequencing only for those with an RNA Integrity Number (RIN) exceeding 5.

### Library preparation and transcriptome sequencing

A NanoPhotometer (IMPLEN) was used to confirm the concentration and purity of RNA. As directed by the manufacturer, using the VAHTS Universal V6 RNA-seq Library Prep Kit for Illumina (Vazyme), we prepared sequencing libraries from qualified total RNAs. The Agilent 2100 Bioanalyzer was used to evaluate the quality of the library, coupled with qPCR-based quantification *via* KAPA Library Quantification Kits. A commercial service provider used an Illumina NovaSeq 6000 platform to sequence pooled libraries.

### Bioinformatic analysis of sequencing data

#### Differential expression analysis

Our bioinformatic pipeline began with quality control and alignment of the raw sequencing reads to the human reference genome GRCh38 using HISAT2. Gene expression was then quantified in FPKM units *via* StringTie. To identify DEGs between the HO and NC groups, we performed statistical analysis with the edgeR package. Genes meeting the dual criteria of an adjusted *p* value (FDR) < 0.05 and a |log2(fold change)| > 2 were designated as significant DEGs.

#### Enrichment Profiling of gene functions

Using the DAVID bioinformatics resource (v6.8), we interrogated the biological significance of the DEGs through profiling of GO terms as well as KEGG pathways. Terms surpassing a significance threshold (*p* value <0.05) were retained for further interpretation.

#### PPI network construction and topological module discovery

Interactions among the proteins encoded by the DEGs (confidence score > 0.7) were predicted using STRING database (version 11.0) and displayed in Cytoscape (version 3.9.1) [23]. Using default parameters, densely connected subnetworks were discovered using the Molecular Complex Detection (MCODE) plugin. Subsequently, the top 20 genes from the most significant module were subjected to further functional profiling *via* the BiNGO plugin, focusing on the enrichment of GO biological processes.

#### Identification of hub genes

To pinpoint central regulators within the PPI network, we employed a multi-algorithm ranking strategy using the cytoHubba plugin in Cytoscape. The highest 10 consensus genes were identified after we ranked nodes using three topological algorithms: Maximum Neighborhood Component (MNC), Degree, and Maximum Clique Centrality (MCC) [24,25].

#### Integrated regulatory network construction

Utilizing the sequencing data of chromatin immunoprecipitation obtained from the ENCODE database *via* the NetworkAnalyst platform, we predicted TFs targeting the promoter regions of DEGs in order to investigate upstream regulators. By combining information from the miRTarBase, TarBase, and miRDB databases, putative microRNA–mRNA interactions were generated. After that, a coordinated TF–microRNA–mRNA regulating network was generated and illustrated in Cytoscape.

### qRT-PCR validation

We selected the four most upregulated and four most downregulated DEGs for qRT-PCR analysis. Utilizing the PrimeScript RT reagent kit (Takara), we synthesized the cDNA. Subsequent qPCR amplification was conducted in triplicate on a QuantStudio 5 system (Applied Biosystems) utilizing SYBR Premix Ex Taq II (Takara). Supplementary Table SI lists the primer sequences. Gene expression levels were normalized to β-actin and analyzed *via* the 2^^(–ΔΔCt)^ method.

### Statistical methods

All statistical analyses were performed using GraphPad Prism software (version 9.0). Differences in continuous variables between the HO and NC groups were assessed with an unpaired, two-tailed Student’s *t* test for normally distributed data; otherwise, the Mann–Whitney *U* test was applied. Categorical variables were compared using Fisher’s exact test. For comparisons of gene expression levels between the HO and NC groups, we employed the method of one-way analysis of variance. In all tests, statistical significance was defined as a *p* value less than 0.05.

## Results

### Clinical data of research cohort

This research cohort comprised four 4 patients with post-traumatic elbow HO and 6 patients who underwent surgery for elbow fractures without HO. The demographic and clinical characteristics of the HO and NC groups are summarized in Table 1. There were no significant differences between the two groups in sex distribution, age, BMI, or the affected side of the elbow joint.

**Table 1.** The demographic characteristics of study patients.

Group	No.	Sex	Age (year)	BMI (kg/m^2^)	Side	HO grade
HO	HO_1	Female	60	22.9	Right	III
	HO_2	Male	35	22.5	Right	IIC
	HO_3	Female	58	20.4	Right	IIC
	HO_4	Male	35	26.8	Right	III
NC	NC_1	Female	69	21.1	Right	–
	NC_2	Male	44	26.0	Left	–
	NC_3	Female	66	27.3	Right	–
	NC_4	Male	19	25.8	Left	–
	NC_5	Female	55	28.7	Right	–
	NC_6	Male	47	20.1	Left	–

BMI, body mass index; HO grade, the Hastings and Graham Classification System for HO at the elbow joint.

### Data normalization and PCA

**Figure 1.**
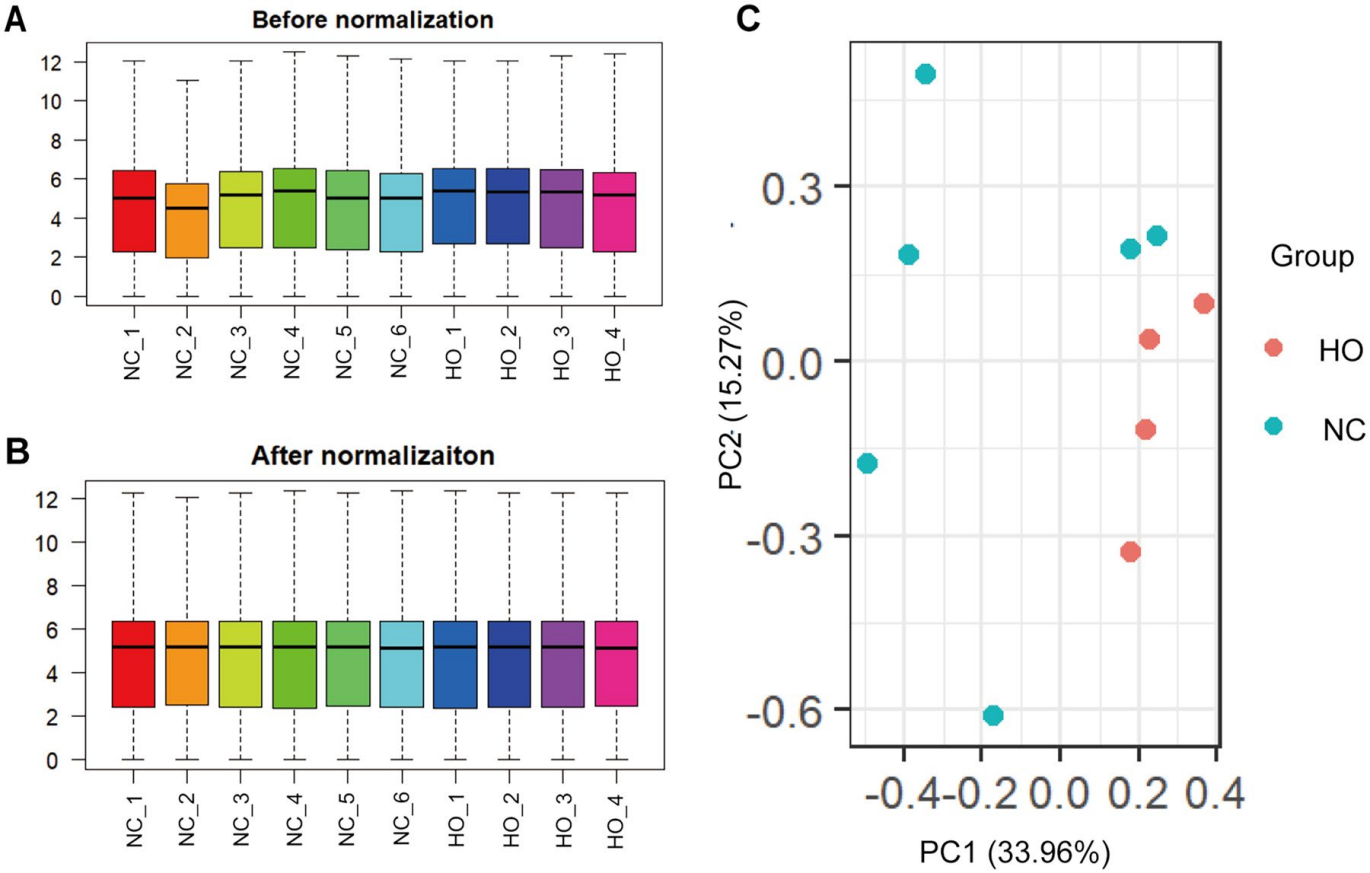
The distribution of expression of all samples before (A) and after (B) normalization. (C) The distribution of expression of samples involving PCA for confirming biological variability between different samples. PCA, principal component analysis.

As shown in Figure 1A and 1B, the black median lines were positioned consistently across all samples after normalization, indicating a high level of standardization and ensuring the reliability of subsequent analyses. The principal component analysis (PCA) plot in Figure 1C shows that samples from the HO and NC groups were clearly separated, reflecting distinct global expression profiles between the two groups.

### Identification of differentially expressed mRNAs and noncoding RNAs

**Figure 2.**
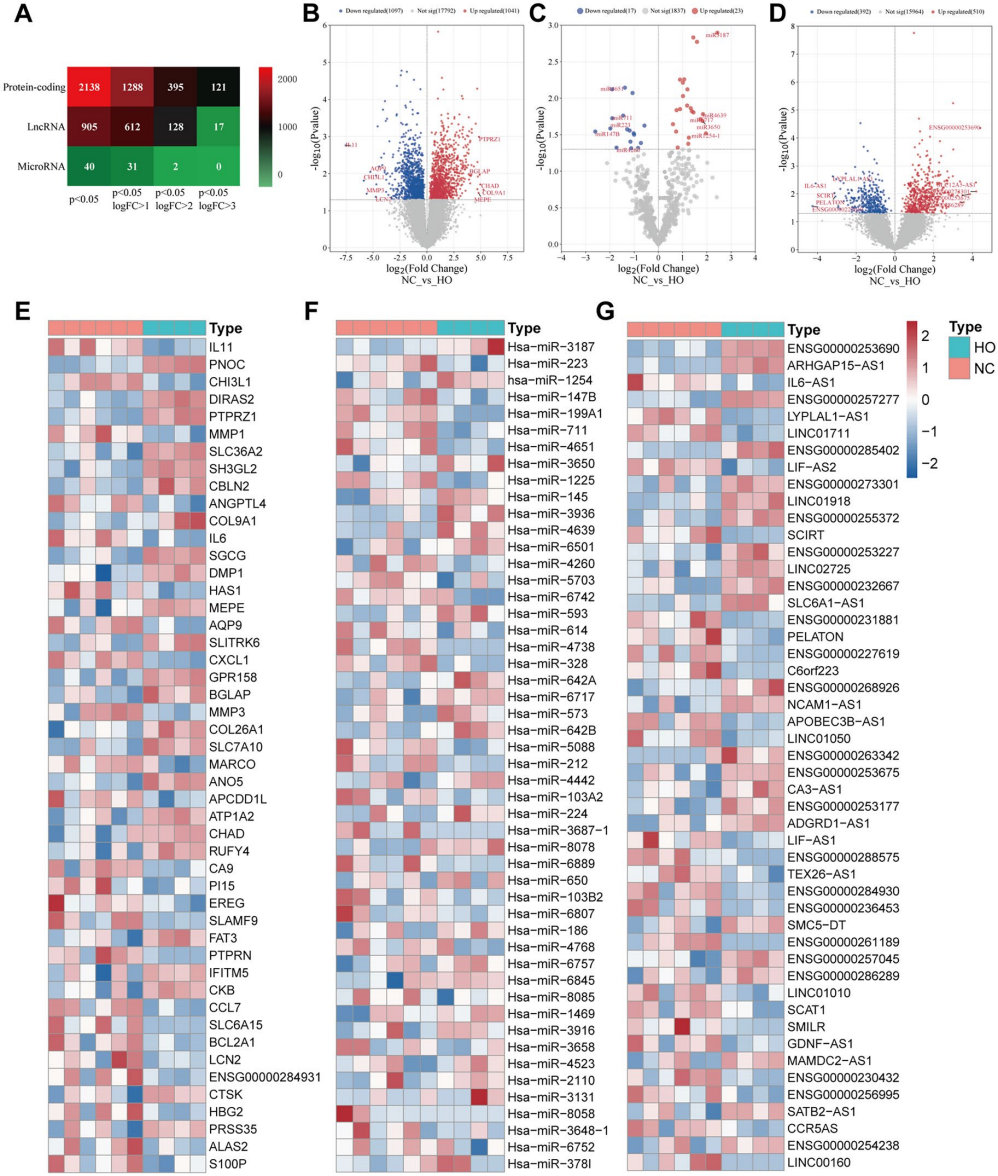
The number of differentially expressed protein-coding genes, lncRNA-coding genes and microRNA-coding genes according to the adjusted *p* value <0.05 and different criteria of logFC (A). The volcano plot show the expression profiles of differentially expressed mRNAs (B), microRNAs (C) and lncRNAs (D). The heatmap show the expression profiles of top 50 differentially expressed mRNAs (E), microRNAs (F) and lncRNAs (G). Red indicates relatively up-regulated, and blue indicates relatively downregulated.

High-throughput sequencing data were analyzed to identify differentially expressed mRNAs and noncoding RNAs based on predefined thresholds. A total of 2,138 DEGs were identified between the HO and NC groups, including 1,041 upregulated and 1,097 downregulated transcripts. In addition, 905 differentially expressed long noncoding RNAs (DELs) were detected, comprising 510 upregulated and 395 downregulated transcripts. Furthermore, 40 differentially expressed microRNAs (DEMs) were identified, with 23 upregulated and 17 downregulated (Figure 2A). The expression profiles of mRNAs, microRNAs, and lncRNAs in both groups are illustrated in the volcano plots (Figure 2B–D). The heatmaps in Figure 2E–G illustrate the top 50 DEGs, DEMs, and DELs. These differential expression profiles provide a basis for subsequent bioinformatics analyses and for elucidating the molecular regulatory mechanisms underlying post-traumatic elbow HO.

### Top ten differentially expressed RNA in HO

**Figure 3.**
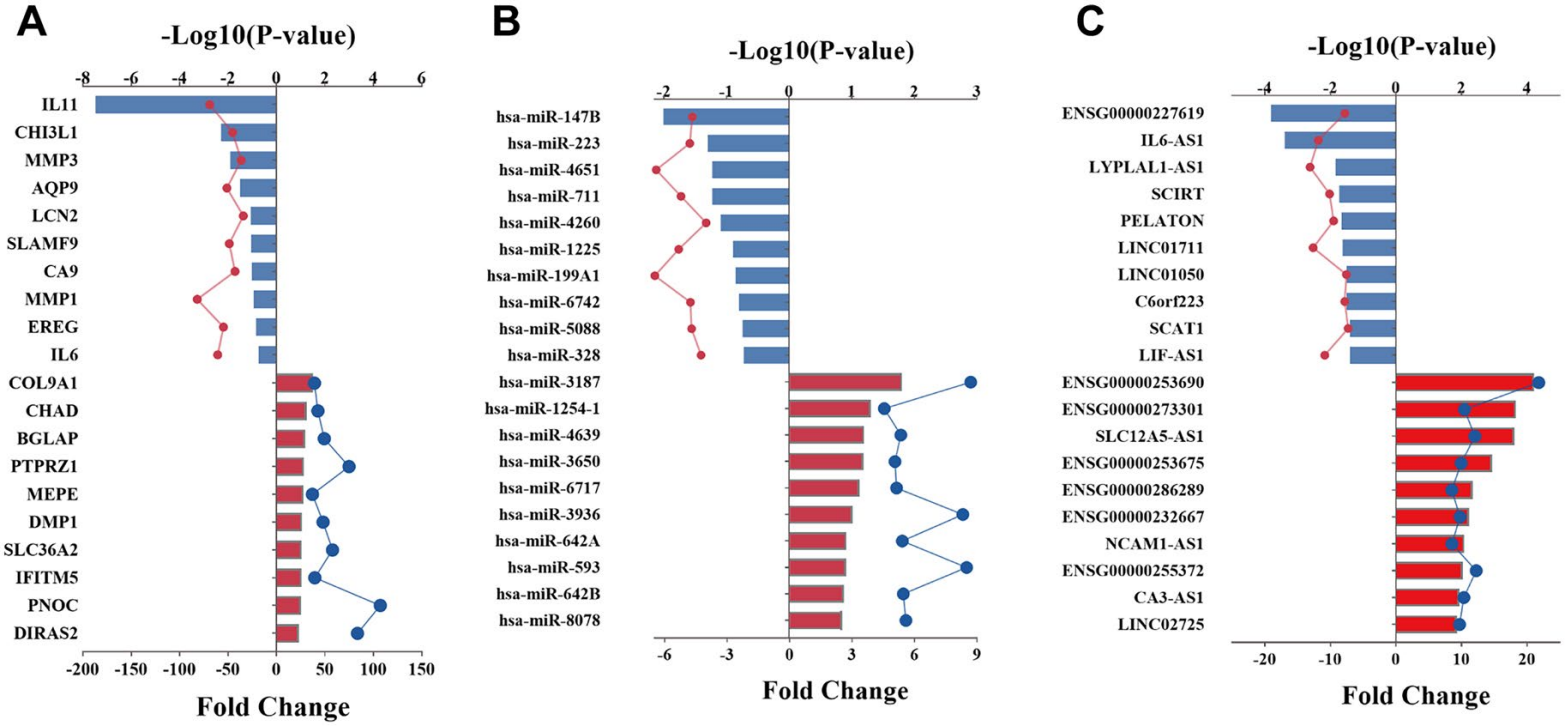
The top ten up- and down-regulated mRNAs (A), microRNAs (B), and lncRNAs (C). Red indicates relatively up-regulated, and blue indicates relatively down-regulated.

The top 10 differentially expressed mRNAs, microRNAs, and lncRNAs are presented in Figure 3. The 10 most upregulated mRNAs were COL9A1, CHAD, BGLAP, PTPRZ1, MEPE, DMP1, SLC36A2, IFITM5, PNOC, and DIRAS2, whereas the 10 most downregulated mRNAs in the HO group were IL11, CHI3L1, MMP3, AQP9, LCN2, SLAMF9, CA9, MMP1, EREG, and IL6.

The most upregulated microRNAs included hsa-miR-3187, hsa-miR-1254-1, hsa-miR-4639, hsa-miR-3650, hsa-miR-6717, hsa-miR-3936, hsa-miR-642a, hsa-miR-593, hsa-miR-642b, and hsa-miR-8078, while the most downregulated microRNAs were hsa-miR-147b, hsa-miR-223, hsa-miR-4651, hsa-miR-711, hsa-miR-4260, hsa-miR-1225, hsa-miR-199a-1, hsa-miR-6742, hsa-miR-5088, and hsa-miR-328.

The 10 most upregulated lncRNAs were ENSG00000253690, ENSG00000273301, SLC12A5-AS1, ENSG00000253675, ENSG00000286289, ENSG00000232667, NCAM1-AS1, ENSG00000255372, CA3-AS1, and LINC02725, whereas the 10 most downregulated lncRNAs were ENSG00000227619, IL6-AS1, LYPTAL1-AS1, SCIRT, PELATON, LINC01711, LINC01050, C6orf223, SCAT1, and LIF-AS1.

### Enrichment Profiling of upregulated DEGs

**Figure 4.**
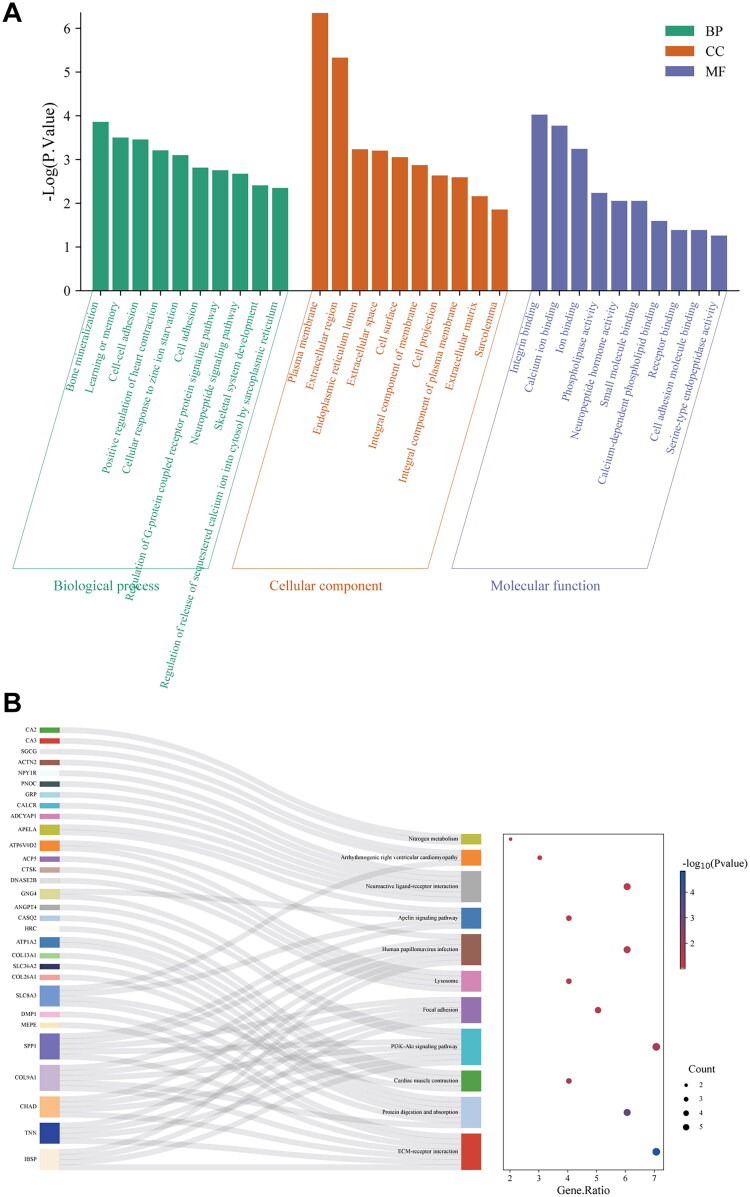
GO enrichment analysis (A) of upregulated DEGs in relevant biological processes, molecular functions, cellular components. KEGG pathways analysis (B) of upregulated DEGs. DEGs, differentially expressed genes; GO, Gene Ontology; KEGG, Kyoto Encyclopedia of Genes and Genomes.

The top 10 enriched GO terms in each category are presented in Figure 4A. Among the biological processes (BPs), the most significantly enriched terms were ‘bone mineralization,’ ‘learning or memory,’ and ‘cell–cell adhesion.’ In the category of cellular components (CCs), the upregulated DEGs were mainly related to ‘plasma membrane’ and ‘extracellular region.’ For molecular functions (MFs), significant enrichment was observed in ‘integrin binding,’ ‘calcium ion binding,’ and ‘ion binding.’

Additionally, a significant correlation between the upregulated differentially expressed genes and 11 signaling pathways, involving extracellular matrix (ECM)–receptor interaction, PI3K–Akt signaling pathway, protein digestion and absorption, human papillomavirus infection, and neural activity ligand–receptor interaction, as shown in the KEGG pathway analysis in Figure 4B.

### Enrichment Profiling of downregulated DEGs

**Figure 5.**
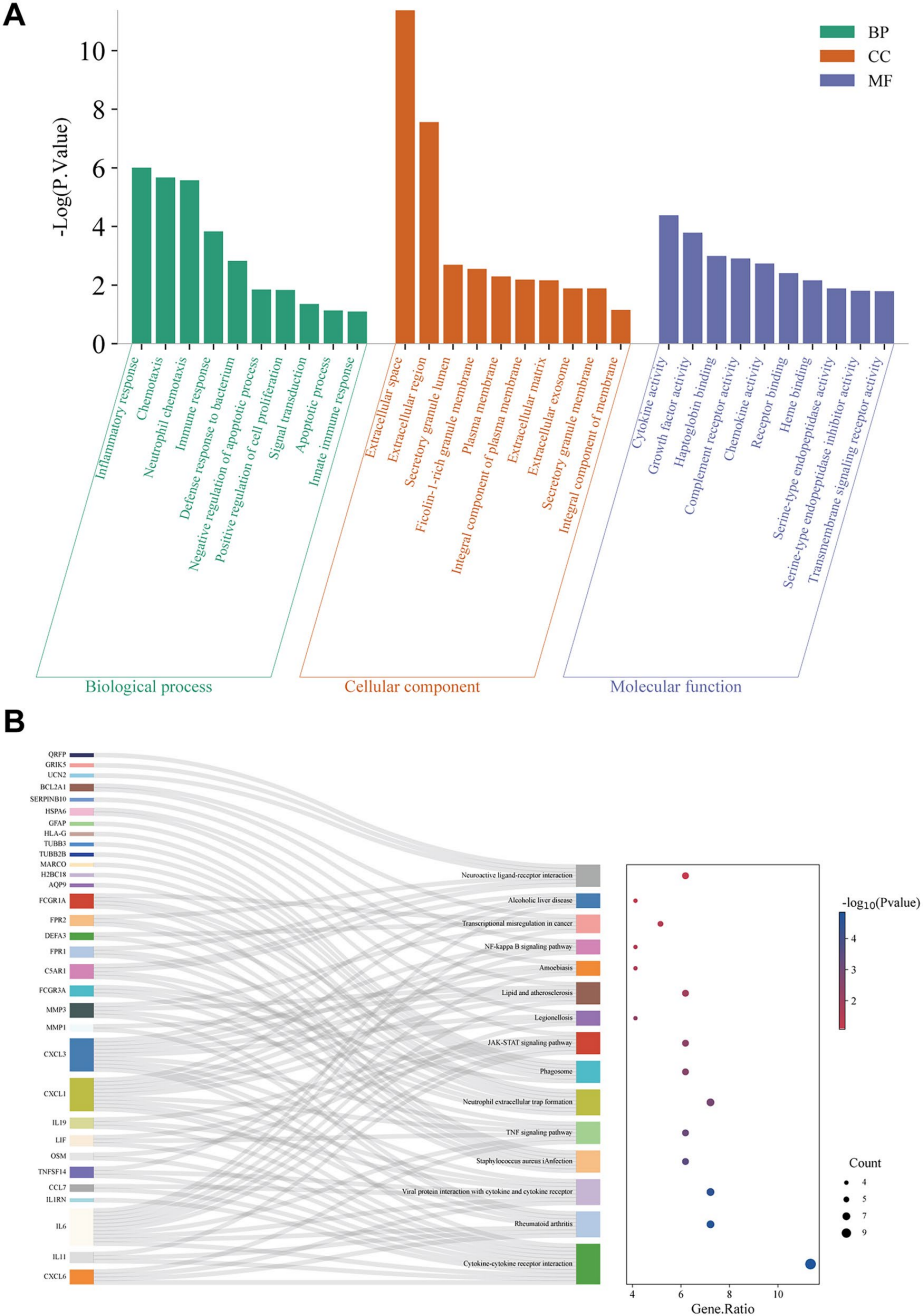
GO enrichment analysis (A) of upregulated DEGs in relevant biological processes, molecular functions, cellular components. KEGG pathways (B) analysis of upregulated DEGs. DEGs, differentially expressed genes; GO, Gene Ontology; KEGG, Kyoto Encyclopedia of Genes and Genomes.

In the GO analysis of Figure 5A, the most significantly enriched BPs among the downregulated DEGs between the HO and NC groups included ‘inflammatory response,’ ‘chemotaxis,’ and ‘neutrophil chemotaxis.’ Within the CC category, these genes were primarily associated with the ‘extracellular space’ and ‘extracellular region.’ For MF, the most significantly enriched terms were ‘cytokine activity’ and ‘growth factor activity.’

Furthermore, cytokine–cytokine receptor interaction, rheumatoid arthritis, viral protein interaction with cytokine and cytokine receptor, and neutrophil extracellular trap formation were among the 15 signaling pathways with which the downregulated DEGs were significantly correlated, according to the KEGG pathway analysis (Figure 5B).

### PPI network construction and topological module discovery

**Figure 6.**
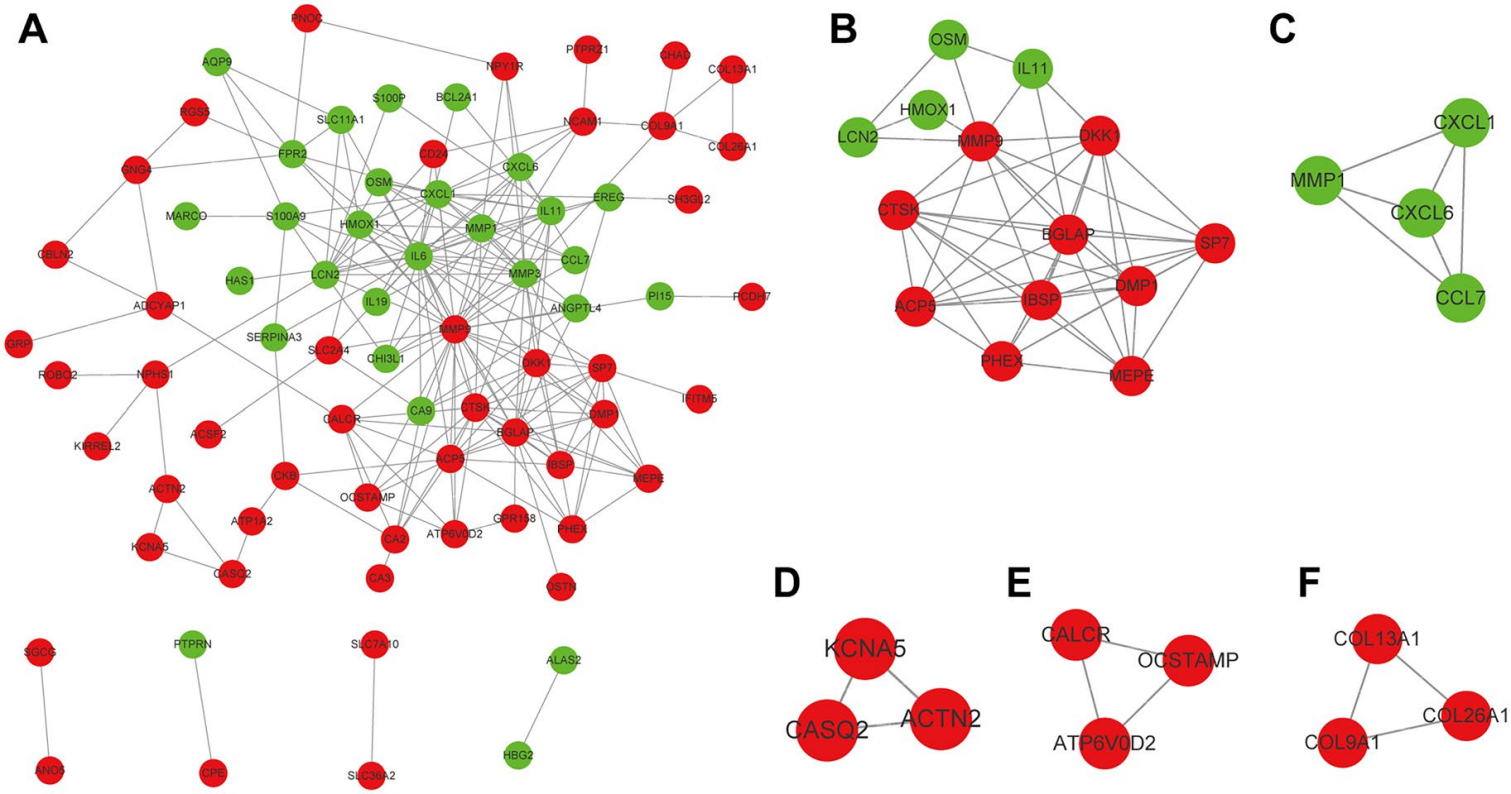
PPI network analysis. (A) The PPI network of DEGs visualized in Cytoscape. Red indicates the upregulated genes, and green indicates the downregulated genes. (B–F) Five significant modules in the PPI network. DEGs, differentially expressed genes; PPI, protein–protein interaction.

The STRING database was used to construct the DEG-based PPI network, which was then shown in Cytoscape. The network comprised 78 nodes and 213 edges (Figure 6A). Among these, the five genes with the highest connectivity degrees (>15) were MMP9 (degree = 30), IL6 (degree = 29), MMP3 (degree = 17), CTSK (degree = 16), and BGLAP (degree = 16), suggesting their potential roles as central regulators in the HO microenvironment.

Subsequently, the MCODE plugin was applied to further explore the PPI network, and five significant modules were discovered (Figure 6B–F), representing highly interconnected subnetworks. Key genes with high MCODE scores within these modules included MMP1, COL9A1, ACTN2, OCSTAMP, and ACP5, all of which may play critical roles in the pathogenesis of post-traumatic HO. Furthermore, the BiNGO plugin was used to explore the biological functions of 20 significantly enriched genes (Figure S1).

### Integrated regulatory network of TF-DEG

**Figure 7.**
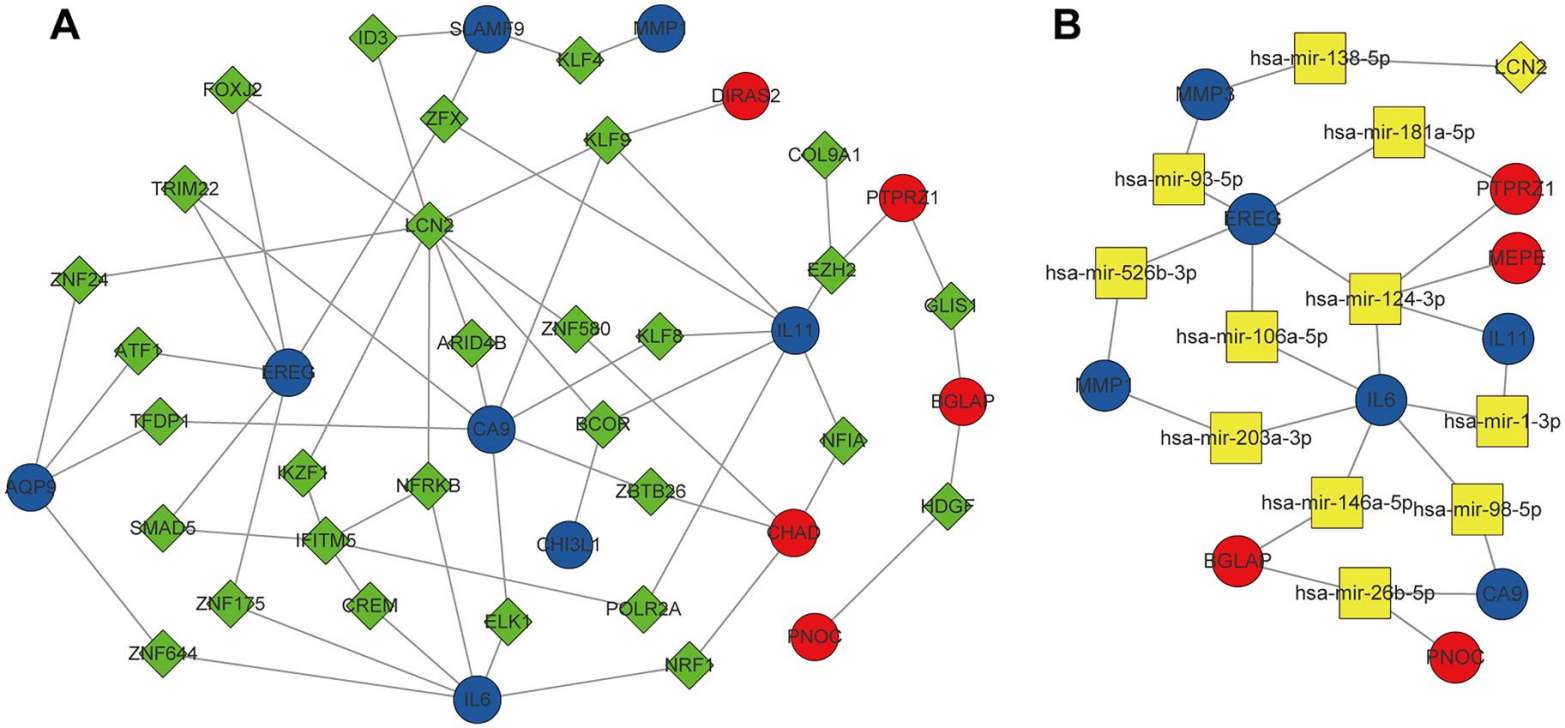
The network of TF–DEG (A) was obtained from the ENCODE database. The network of microRNA–DEG (B) was obtained from the TarBase, miRTarBase, and miRecords databases. The circles indicates DEGs (red indicates upregulated genes, and blue indicates the downregulated genes), the diamonds indicates TFs, and the squares indicates microRNAs. ENCODE, Encyclopedia of DNA Elements; TF, transcription factor; DEG, differentially expressed gene.

To clarify the transcriptional control of specific DEGs, a prospective TF-DEG interaction network has been developed using genomic coordinate information and TF binding domain data. Thirteen DEGs and thirty TFs were discovered to have seventy-three projected regulatory connections. Among them, KLF9 was predicted to regulate three DEGs (CA9, IL11, and DIRAS2), while ZFX was also predicted to regulate three downregulated DEGs (SLAMF9, EREG, and IL11). The overall regulatory network is illustrated in Figure 7A, highlighting potential transcriptional regulatory mechanisms underlying gene dysregulation in post-traumatic elbow HO.

### Integrated regulatory network of microRNA–DEG

MicroRNA-DEG interactions were found by network modeling in order to investigate post-transcriptional regulation processes. This analysis revealed 26 predicted interactions involving 13 microRNAs and 10 DEGs. The resulting network was visualized using Cytoscape (Figure 7B). Among the key findings, IL6 was regulated by six microRNAs—hsa-miR-124-3p, hsa-miR-106a-5p, hsa-miR-203a-3p, hsa-miR-146a-5p, hsa-miR-98-5p, and hsa-miR-1-3p. Notably, hsa-miR-124-3p emerged as a hub microRNA, targeting five DEGs—EREG, IL6, IL11, MEPE, and PTPRZ1—suggesting its potential central role in the post-transcriptional regulation of genes associated with post-traumatic elbow HO.

### TF–microRNA interaction network

**Figure 8.**
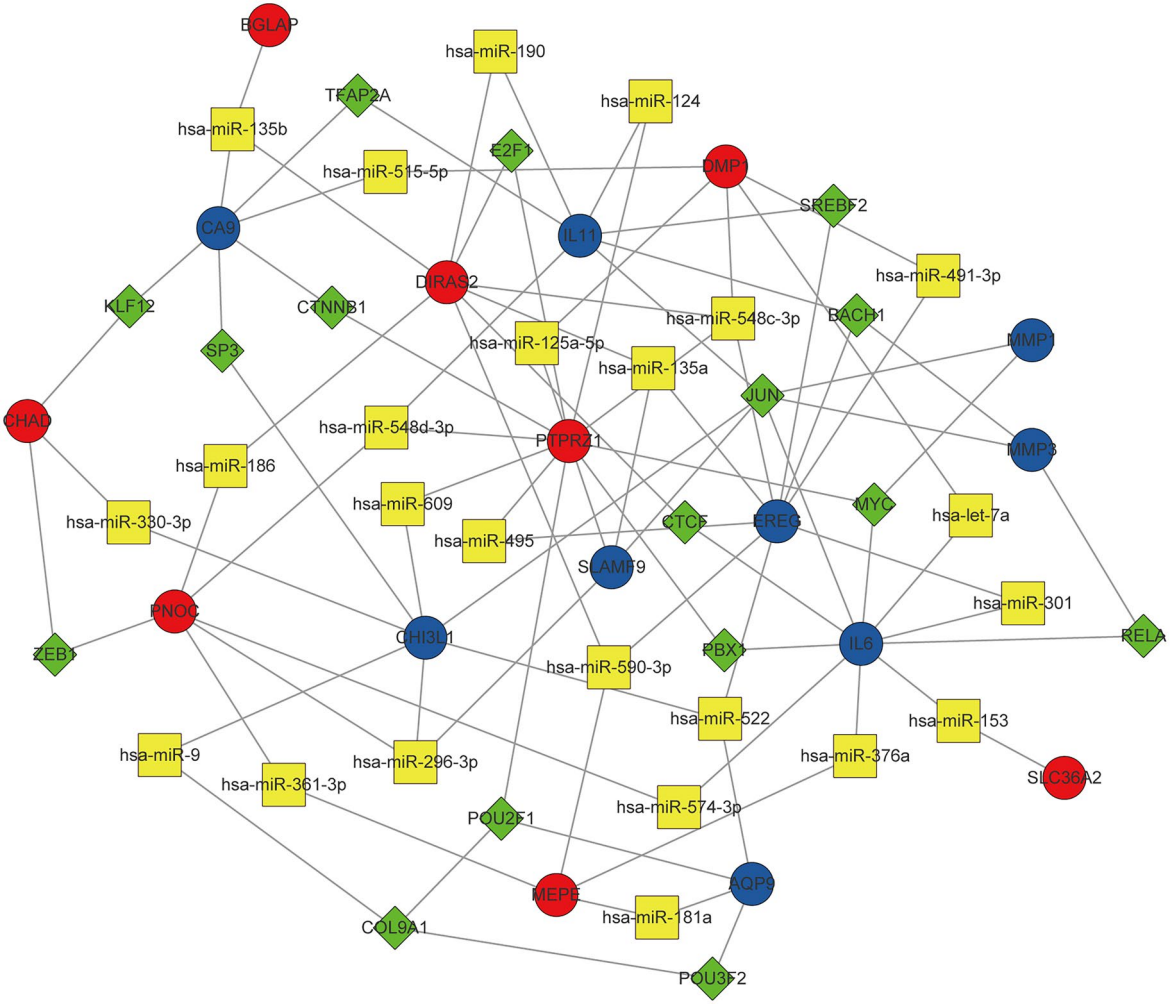
Integrative regulatory network of TF-microRNA. The circles indicates DEGs (red indicates upregulated genes, and blue indicates the downregulated genes), the diamonds indicates TFs, and the squares indicates microRNAs. TF, transcription factor; DEG, differentially expressed gene.

An integrated TF–microRNA interaction network was constructed based on 20 DEGs using Cytoscape (Figure 8). This network comprised 17 DEGs, 16 TFs, and 24 microRNAs, encompassing 70 TF–DEG and 19 microRNA–DEG associations. The connectivity degree of the 17 DEGs was analyzed within both the TF–DEG and microRNA–DEG networks. Notably, the TF JUN regulated six DEGs (IL11, CHI3L1, SLAMF9, IL6, MMP3, and MMP1), indicating its key regulatory role. Additionally, hsa-miR-548c-3p and hsa-miR-135b each regulated four DEGs, suggesting their significant involvement in post-transcriptional regulation within this integrated network.

### Assessment of qRT-PCR

**Figure 9.**
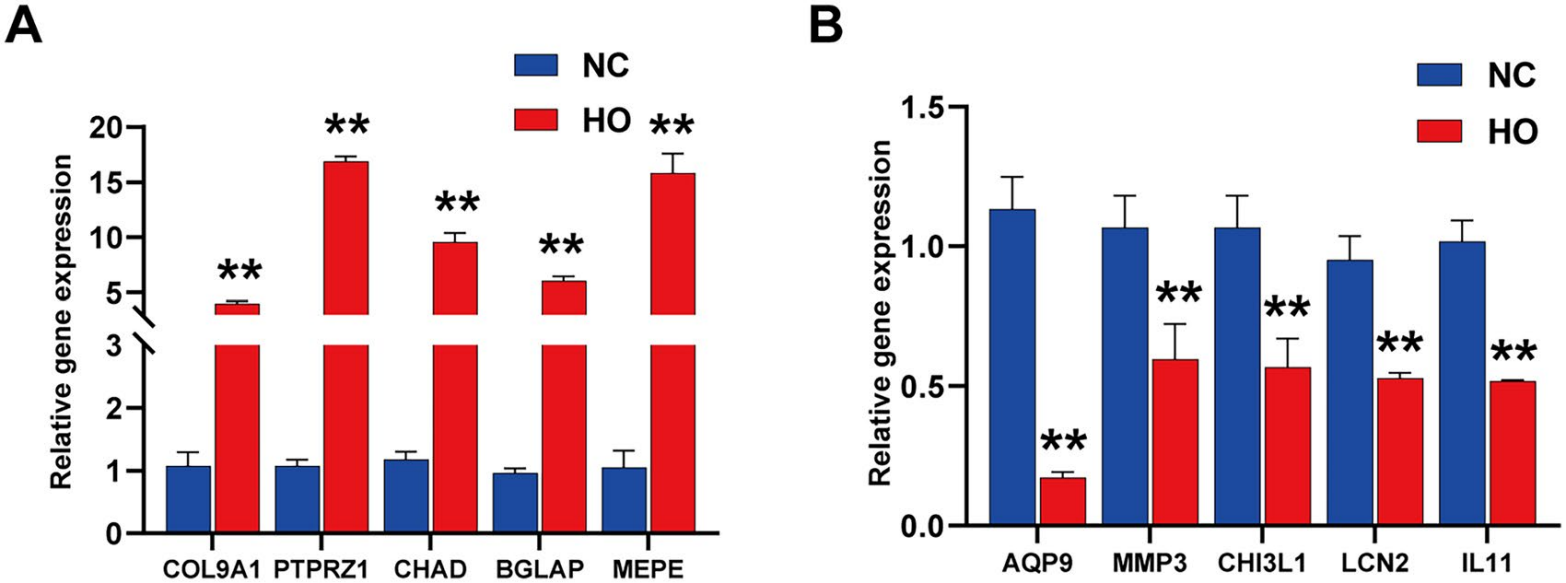
qRT-PCR analysis of the expression levels of upregulated DEGs (A). qRT-PCR analysis of the expression levels of downregulated DEGs (B). **p < 0.001. DEG, differentially expressed gene; qRT-PCR, real-time quantitative reverse transcription polymerase chain reaction.

The five most upregulated and five most downregulated genes were subjected to qRT-PCR in order to further validate the RNA-seq results (Figure 9). The qRT-PCR findings revealed that, compared with the NC group, the expression levels of all ten mRNAs were significantly altered in the HO group, consistent with the high-throughput sequencing results.

## Discussion

Post-traumatic elbow HO can cause local pain and markedly restrict elbow joint mobility, substantially impairing patients’ quality of life [7]. In severe cases, it may lead to complete ankylosis, necessitating surgical release of the joint and imposing a considerable medical and socioeconomic burden [8]. Understanding the specific molecular mechanisms underlying the initiation and progression of post-traumatic elbow HO is essential for developing effective preventive and therapeutic strategies. However, current research in this field remains limited, resulting in a lack of clinically effective treatment options [15–17].

The specific pathological mechanisms that underlie the onset and progression of diseases may be thoroughly investigated using RNA-seq technology [18]. To the best of our knowledge, no previous studies have reported RNA-seq data specifically for post-traumatic elbow HO. Thus, utilizing high-throughput RNA-seq, the current work offers the first thorough examination of mRNA, microRNA, and lncRNA expression levels in post-traumatic elbow HO compared with normal bone tissue.

In total, we identified 2,138 DEGs between the HO and NC groups, including 1,041 upregulated and 1,097 downregulated genes. Additionally, 905 DELs were detected, comprising 510 upregulated and 395 downregulated. Furthermore, 40 DEMs were identified, with 23 upregulated and 17 downregulated. The ten most upregulated mRNAs were COL9A1, CHAD, BGLAP, PTPRZ1, MEPE, DMP1, SLC36A2, IFITM5, PNOC, and DIRAS2, whereas the ten most downregulated mRNAs included IL11, CHI3L1, MMP3, AQP9, LCN2, SLAMF9, CA9, MMP1, EREG, and IL6. Among these, IL11 showed the greatest degree of upregulation. IL-11, a cytokine belonging to the IL-6 family, mainly signals *via* the classical IL-11/IL-11 receptor (IL-11R)/glycoprotein 130 (gp130) pathway [26]. Numerous studies have examined IL-11’s regulatory function in bone metabolism, particularly regarding its effects on osteogenesis and osteoclastogenesis [26–29]. Recent studies have expanded this understanding, revealing that IL-11 influences multiple bone-related cell types, including osteoblasts, osteoclasts, bone marrow stromal cells, adipose tissue-derived mesenchymal stem cells, and chondrocytes. Collectively, IL-11 contributes to bone homeostasis by regulating osteogenesis, osteolysis, bone marrow hematopoiesis, adipogenesis, and even bone metastasis.

Another gene of interest, CHI3L1 (chitinase 3-like 1), a member of the glycoside hydrolase 18 family, has been shown to regulate osteoclast differentiation and bone resorption [30–34]. Xu et al. [30] demonstrated that CHI3L1 acts through its receptor IL13Rα2 to enhance RANKL-induced activation of the MAPK and AKT signaling pathways, thereby promoting osteoclast differentiation. In addition, CHI3L1 has been reported to stimulate bone formation, inhibit osteoclastogenesis, and protect against age-related osteoporosis [35].

In our study, several differentially expressed genes—including matrix metalloproteinases (MMPs)—were identified. Although MMPs are commonly involved in inflammation and physiological bone remodeling, their dysregulation in this context likely reflects a specific role in HO rather than a nonspecific injury response. Increasing evidence supports this hypothesis: MMP9, for example, functions as an early HO-specific marker, defining the ectopic niche rather than general wound regions [36,37], and its expression correlates with HO progression [38]. Genetic deletion of MMP9 has been shown to reduce HO formation in fibrodysplasia ossificans progressiva models [39], confirming its pathogenic involvement. Moreover, other MMPs, such as MMP10, can modulate bone morphogenetic protein (BMP) signaling [40], further implicating this family in HO pathogenesis. Collectively, MMPs appear to sustain a chronic pro-osteogenic microenvironment and disrupt local tissue homeostasis, promoting the differentiation of progenitor cells into osteoblasts at ectopic sites. These findings suggest that MMPs act not only as inflammatory mediators but also as potential HO-specific therapeutic targets, whose inhibition may prevent pathological ossification without impairing normal bone repair.

Our transcriptomic analysis revealed significant upregulation of interleukin-6 (IL-6) in HO tissues. Beyond its established role as a proinflammatory cytokine, IL-6 appears to directly contribute to pathological osteogenesis. For instance, IL-6 secreted by M1 macrophages has been shown to promote osteogenic differentiation in ligamentum flavum cells [41]. In addition, activation of the IL-6/STAT3 signaling pathway contributes mechanistically to HO by suppressing miR-135b, which in turn downregulates BMPER, a negative regulator of BMP signaling, thereby facilitating aberrant bone formation [42]. Clinically, elevated IL-6 levels have been correlated with ossification severity [43,44]. Persistent IL-6 signaling may therefore serve as a distinguishing feature of pathological HO relative to normal bone repair. Thus, IL6 likely functions as a specific pathogenic driver of HO rather than a nonspecific inflammatory byproduct, making it a promising therapeutic target for preventing ectopic bone formation without impairing physiological healing.

GO and KEGG pathway analyses of the DEGs in post-traumatic elbow HO revealed their involvement in several key biological processes and pathways, including bone mineralization, plasma membrane organization, and integrin binding. The PI3K–Akt, NF-κB, JAK–STAT, and TNF signaling pathways were among the critical signaling pathways in which these DEGs were significantly enriched. Our sequencing results further confirmed the central role of the PI3K–Akt pathway in HO, aligning with existing literature that implicates this pathway in both osteogenesis and inflammation—two essential processes in HO development. In osteogenesis, PI3K–Akt activation by osteogenic factors such as BMP2 promotes osteoblast differentiation through the suppression of PTEN [45,46]. In the context of inflammation, PI3K–Akt interacts with signaling pathways such as NF-κB and contributes to macrophage-mediated HO through neurotrophin-3-dependent mechanisms [47–49]. Importantly, PI3K–Akt functions as a central signaling hub, integrating inputs from multiple HO-inducing stimuli—including NRG3/ErbB3 signaling during denervation or injury-induced stress—into a unified pro-osteogenic response [49–51]. Therefore, PI3K–Akt not only bridges inflammatory activation and osteogenic differentiation in HO but also represents a promising dual-action therapeutic target capable of concurrently attenuating both mechanisms.

Our transcriptomic data revealed significant activation of the NF–κB signaling pathway in HO tissues, reinforcing its central role in HO pathophysiology [47,52]. Beyond its canonical inflammatory function, NF–κB serves as a critical molecular link that converts upstream triggers—such as trauma or ACVR1 mutations—into downstream osteogenic responses [52,53]. It promotes a proinflammatory and pro-osteogenic microenvironment by upregulating cytokines such as TNF-α and IL-6, and by interacting with signaling cascades including mTORC1 and MAPK, thereby driving chondrogenic and osteogenic differentiation [54,55]. This activation induces the expression of osteogenic transcription factors such as RUNX2, which direct mesenchymal progenitor cells toward bone formation [54]. Notably, pharmacological inhibition of NF–κB has been shown to reduce HO formation in animal models [56–58], underscoring its therapeutic relevance. Collectively, our findings highlight NF–κB–associated genes as key mediators and potential molecular targets in the pathogenesis of HO.

The JAK–STAT signaling pathway played a crucial role in HO, as our sequencing findings demonstrated. This pathway serves as a key downstream effector of injury-induced inflammatory cytokines such as IL-6, amplifying inflammation while directly promoting the osteogenic differentiation of progenitor cells [59,60]. Supporting this notion, Alexander et al. demonstrated that pharmacological inhibition of JAK1/2 using ruxolitinib significantly reduced HO formation following spinal cord injury [61]. Furthermore, dysregulation of SOCS3, a physiological inhibitor of JAK–STAT signaling, has been associated with ligamentum flavum ossification [62]. Within this mechanistic framework, our findings position JAK–STAT signaling as a pivotal nexus that converts transient inflammatory cues into sustained osteogenic activation—thereby representing a promising therapeutic target for the prevention of HO.

TFs and microRNAs are recognized as key regulators of bone homeostasis and play pivotal roles in the pathogenesis of bone disorders, including HO [63,64]. To investigate these regulatory interactions in post-traumatic elbow HO, a coordinated TF-miRNA–mRNA regulating network was constructed. The network revealed that the transcription factor JUN regulates six DEGs—IL11, CHI3L1, SLAMF9, IL6, MMP3, and MMP1—indicating its central role in the initiation and progression of HO following elbow trauma. Along with c-Fos, Fra1, Fra2, JunB, and JunD, JUN is a member of the transcription factor family known as activator protein-1 (AP-1) [65–67]. JUN has been implicated in fibrotic pathologies and is known to regulate essential cellular processes such as proliferation and the cell cycle [68,69]. Notably, Lerbs et al. [70] demonstrated that JUN activates hedgehog signaling in skeletal stem cells, promoting bone formation by expanding osteoprogenitor populations and directing them toward an osteogenic lineage. Collectively, these findings position JUN as a promising molecular target for the prevention and treatment of post-traumatic elbow HO.

Regarding microRNAs, hsa-miR-124-3p emerged as the most highly connected node in the regulatory network, targeting five DEGs: EREG, IL6, IL11, MEPE, and PTPRZ1. Additionally, hsa-miR-548c-3p and hsa-miR-135b each regulated four DEGs. Previous studies [71,72] have demonstrated that miR-124 is downregulated during the osteogenic differentiation of bone marrow-derived mesenchymal stem cells and plays a crucial regulatory role in this process. For instance, Sp7, a key transcription factor required for osteoblast differentiation, has been identified as a direct target of miR-124 [73]. Furthermore, Qadir et al. [74] reported that BMP2 suppresses miR-124 expression, which functions as a negative regulator of osteoblast differentiation by targeting the Dlx family of transcription factors (Dlx5, Dlx3, and Dlx2). Functionally, inhibition of miR-124 accelerates osteogenic differentiation and enhances bone formation *in vivo*.

miR-135b has also been implicated in osteogenic regulation, modulating the differentiation of mesenchymal stromal cells (MSCs) under pathological conditions such as multiple myeloma [75]. Furthermore, miR-135b may inhibit osteogenic differentiation and osteoblast proliferation by targeting RUNX2, thereby contributing to the onset and progression of osteoporosis [76]. In addition, miR-135a, which shares close sequence homology with miR-135b, acts as a suppressor of osteogenic differentiation in senescent vascular smooth muscle cells by regulating the KLF4/STAT3 pathway [77]. Bioinformatics analyses have predicted putative binding sites between miR-135b and BMPER (bone morphogenetic protein endothelial cell precursor-derived regulator), a key mediator that promotes osteogenesis-angiogenesis coupling in human bone-derived MSCs [42]. Moreover, RNA interference–mediated knockdown of BMPER inhibits osteoblast-like differentiation in human coronary artery smooth muscle cells, underscoring its essential role in osteogenesis [78,79].

Our integrated TF–mRNA–miRNA network analysis identified key regulatory hub molecules, including the transcription factor JUN, microRNAs (hsa-miR-124-3p, hsa-miR-548c-3p, hsa-miR-135b), and critical genes (MMP9, IL6, MMP3, CTSK, BGLAP). These elements appear to form a coherent regulatory circuit that aids in the onset and progression of HO by coordinating inflammatory signaling, ECM remodeling, and terminal osteogenic differentiation. At the center of this network lies JUN, a pivotal component of the AP-1 complex that is activated by diverse cellular stressors. The predicted upregulation of JUN likely acts as a master transcriptional switch, promoting the expression of proinflammatory and matrix-degrading mediators such as IL6 and MMP9. This JUN/IL6/MMP9 regulatory axis may establish a feed-forward loop, wherein IL6 sustains a local inflammatory milieu while MMP9, together with MMP3, mediates ECM degradation. These processes collectively generate a permissive microenvironment for the recruitment and infiltration of osteoprogenitor cells. The stability of this pro-osteogenic signaling program appears to be fine-tuned by specific microRNAs. Downregulation of hsa-miR-124-3p, a predicted upstream inhibitor of JUN, could remove a critical repressive checkpoint, thereby amplifying JUN-driven transcriptional activation. Concurrently, dysregulation of hsa-miR-135b, which may target MMP9, could further disrupt ECM homeostasis, facilitating aberrant tissue remodeling. As HO progresses, the network shifts toward bone maturation, reflected by the involvement of CTSK in bone resorption and the pronounced expression of BGLAP, a late-stage osteoblast marker indicative of bone mineralization. In summary, our proposed model suggests that early HO triggers activate JUN, whose downstream effects are amplified by the loss of repressive microRNAs, leading to persistent inflammation and ECM degradation that ultimately culminate in ectopic bone matrix deposition. This mechanistic framework offers experimentally testable hypotheses and highlights potential regulatory nodes for targeted therapeutic intervention in post-traumatic elbow HO.

While this integrated bioinformatics study provides novel insights into the molecular network of post-traumatic elbow HO, several limitations should be acknowledged. Firstly, the relatively small sample size of the RNA-seq dataset, although sufficient for initial discovery, may limit the statistical power and generalizability of our findings. This constraint is common in the study of rare clinical conditions but necessitates cautious interpretation. Secondly, and more critically, the study is inherently restricted by its computational nature and lacks direct functional validation for the identified hub genes, microRNAs, and TFs. To address these limitations and translate our predictions into biological understanding, we propose a clear roadmap for future research. Immediate next steps should include validating the differential expression of key targets using larger, independent clinical cohorts or established animal models of HO through methods like qRT-PCR and immunohistochemistry. Subsequently, the biological roles of the most promising candidates key hub genes must be tested *in vitro* using gain- and loss-of-function experiments in relevant progenitor cells, assessing their impact on osteogenic differentiation and key pathway activity. Ultimately, *in vivo* interventional studies in HO models, such as local modulation of key microRNAs or genes, will be crucial to confirm their mechanistic involvement and therapeutic potential. These sequential validation efforts will be essential to solidify the reliability of our findings and bridge the gap between predictive bioinformatics and clinical application.

## Conclusions

In summary, to the best of our knowledge, this study is the first to comprehensively characterize the expression profiles of mRNAs, microRNAs, and lncRNAs in post-traumatic elbow HO using high-throughput RNA-seq. Our findings provide novel insights into the molecular mechanisms underlying HO development. The identified hub genes—MMP9, IL6, MMP3, CTSK, and BGLAP—are closely associated with inflammation, extracellular matrix remodeling, and osteogenic differentiation. In addition, key regulatory molecules, including the transcription factor JUN and microRNAs (hsa-miR-124-3p, hsa-miR-548c-3p, and hsa-miR-135b), may represent promising targets for future diagnostic, preventive, and therapeutic interventions in post-traumatic elbow HO.

## Supplementary Material

Supplementary material.docx

## Data Availability

The datasets used and/or analyzed during the current study are available from the corresponding author on reasonable request.
