# Supplementary material for: Comprehensive analysis of mRNA–microRNA–lncRNA expression profiles in post-traumatic elbow heterotopic ossification using RNA sequencing and experimental validation
Source: Ann Med. 2026 Jan 13;58(1):2611612. doi: 10.1080/07853890.2025.2611612 (PMC12805848; doi:10.1080/07853890.2025.2611612)

| Table SI. The primers used in qRT-PCR | | |
| --- | --- | --- |
| Gene name | Primer | Sequence (5'-3') |
| Homo-COL9A1-70 | Forward | GACGGTGACCGAGGTCCTA |
|  | Reverse | CTCCTGGCTTTCCCGGTTC |
| Homo-PTPRZ1-105 | Forward | TGTGACTGAACTGCCACCTC |
|  | Reverse | GCAGTCCCCGACAAGTTCAT |
| Homo-CHAD-193 | Forward | GTCCCACAACCCCCTGAAAA |
|  | Reverse | GTCGAAGGGGAAGTTGGAGG |
| Homo-BGLAP(OCN)-133 | Forward | TCACACTCCTCGCCCTATTG |
|  | Reverse | CTCTTCACTACCTCGCTGCC |
| Homo-MEPE-168 | Forward | GTTTGCAGGCCCAAGTGAAG |
|  | Reverse | TCGTTGCTGCCCTCTACAAG |
| Homo-AQP9-75 | Forward | CCTTTGGTTGGTGCTGTCATT |
|  | Reverse | TGAGTCAGGCTCTGGATGGTG |
| Homo-MMP3-156 | Forward | TGGACAAAGGATACAACAGGGAC |
|  | Reverse | ATCTTGAGACAGGCGGAACC |
| Homo-YKL40(CHI3L1)-108 | Forward | TGTTCCGAGGTCAGGAGGAT |
|  | Reverse | TGCCCATCACCAGCTTACTG |
| Homo-LCN2-101 | Forward | CTACGGGAGAACCAAGGAGC |
|  | Reverse | GGACAGGGAAGACGATGTGG |
| Homo-IL11-100 | Forward | TCCACTTGAGGGCGATTTGT |
|  | Reverse | CTCGGGACCTCCACCTGAAT |
| Homo-β-actin-250 | Forward | CATGTACGTTGCTATCCAGGC |
|  | Reverse | CTCCTTAATGTCACGCACGAT |

Figure S1. Biological process analysis of the top 20 differentially expressed genes was constructed using BiNGO. The color depth of nodes refers to the corrected p-value of ontologies. The size of nodes refers to the number of genes that are involved in the ontologies. P < 0.001 was considered statistically significant.


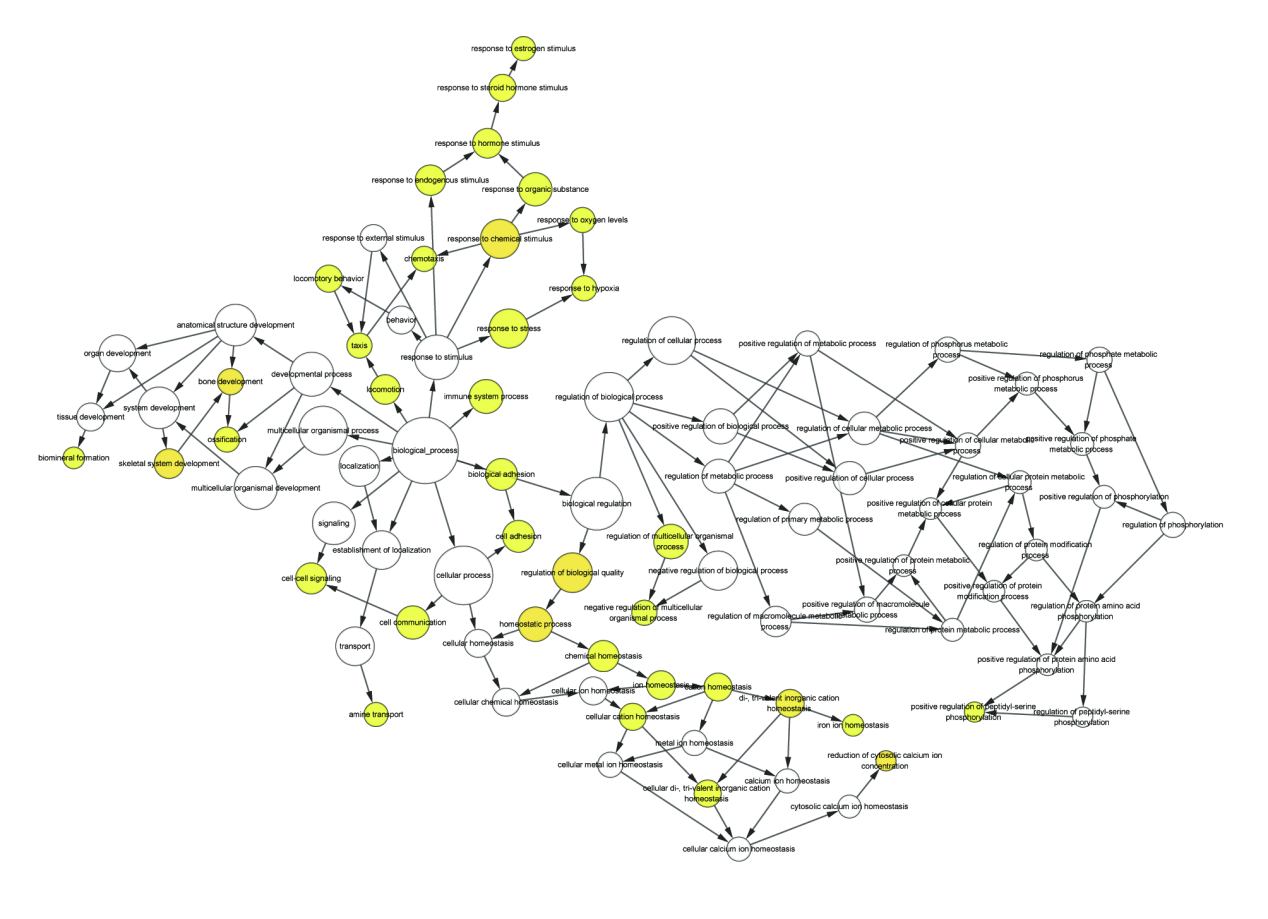

Supplement: Supplementary material.docx [file IANN_A_2611612_SM7396.docx]
